# Islet Proteome Remodeling and Proteostasis Disruption in HFSC-Fed Mice

**DOI:** 10.1021/acsomega.6c00395

**Published:** 2026-05-05

**Authors:** Vijayalakshmi Gangadhara, Yalpi Karthik, Ravichandran Manisekaran, Asha Abraham

**Affiliations:** † Father George Albuquerque Pai Cell and Molecular Biology Laboratory, Department of Biotechnology, School of Life Sciences, St Aloysius (Deemed to be University), Mangaluru 575003, Karnataka, India; ‡ Agrogenomic Sciences, National School of Higher Studies Unit-León, National Autonomous University of Mexico (UNAM), C.P. 37689 León, Guanajuato, Mexico; § Interdisciplinary Research Laboratory (LII), Nanostructures and Biomaterials Area, Escuela, Nacional de Estudios Superiores Unidad León, Universidad Nacional Autónoma de México (UNAM), Predio el Saucillo y el Potrero, Comunidad de los Tepetates, León C.P. 37684, Mexico

## Abstract

Chronic overnutrition promotes metabolic syndrome and imposes substantial stress on pancreatic tissue, yet the early molecular alterations within pancreatic islets remain incompletely understood. High-fat, simple-carbohydrate (HFSC) diets have previously been shown to induce pancreatic steatosis, acinar atrophy, and islet hypertrophy in C57BL/6J mice. To investigate molecular responses underlying HFSC-induced pancreatic remodeling, we analyzed proteomic alterations in collagenase-isolated pancreatic islets from male C57BL/6J mice fed a laboratory-formulated HFSC diet for 150 days (*n* = 3 per group). Islet proteins were profiled using label-free ESI-nanoLC-MS/MS, resulting in the identification of 386 proteins. After applying peptide-based filtering criteria (≥2 peptides, ≥1 unique peptide) and statistical thresholds (fold change ≥ 1.5 and false discovery rate (FDR)-adjusted *q*-value ≤ 0.05), 30 proteins were identified as differentially expressed, including 19 upregulated and 11 downregulated proteins in HFSC islets compared with controls. Functional enrichment analysis revealed significant upregulation of pathways related to cytoplasmic translation, ribosomal biogenesis, and endoplasmic reticulum (ER) protein-folding processes, indicating increased biosynthetic activity and activation of proteostasis mechanisms. In contrast, downregulated proteins were enriched in carbohydrate and lipid metabolic pathways, suggesting metabolic remodeling within pancreatic islets. Notably, stress-responsive proteins, including MANF, HYOU1, SDF2L1, and HSP90B1, were downregulated, supporting the presence of ER stress-associated proteostasis alterations under HFSC-dietary conditions. Together, these findings provide proteomic evidence of molecular adaptations associated with metabolic stress in pancreatic islets, highlighting potential pathways involved in early islet dysfunction during diet-induced metabolic syndrome.

## Graphical abstract

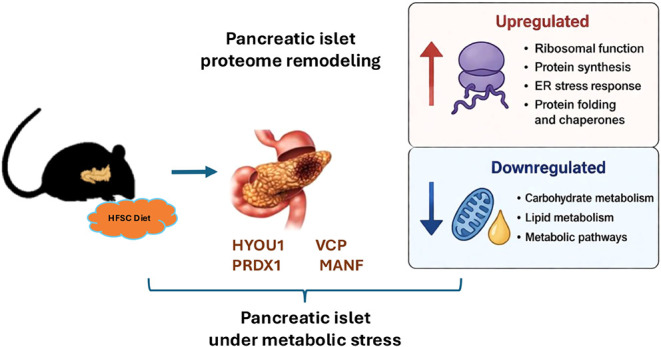

## 1 Introduction

Metabolic syndrome is characterized by insulin resistance, hyperglycemia, dyslipidemia, and visceral obesity, and significantly increases the risk of type 2 diabetes and cardiovascular disease.^1^ While metabolic stress broadly disrupts multiple organs, the pancreas plays a central role in maintaining glucose homeostasis and is therefore highly vulnerable to chronic nutrient excess and metabolic overload.^2^ Ectopic fatty deposits in the pancreas, also known as nonalcoholic fatty pancreatic disease (NAFPD), are getting recognized as a major pathogenic factor to poor endocrine function.^3^ β-Cell hyperplasia and compensatory hyperinsulinemia support normoglycemia in the early phases of metabolic syndrome.^4^

Persistent metabolic stress causes oxidative and endoplasmic reticulum (ER) stress, inflammation, and β-cell malfunction, ultimately impairing glucose control. Although the functional changes that occur in the pancreas during metabolic syndrome^5^ are well-known, the early cellular remodeling events and molecular modifications inside pancreatic islets are poorly understood. Most available studies employ systemic metabolic data or whole-tissue analysis, which do not capture islet-specific proteome changes that may represent early processes before the onset of severe diabetes.^6,7^ To better simulate human dietary patterns that contribute to metabolic syndrome, we have developed a high-fat simple-carbohydrate (HFSC) diet model in C57BL/6J mice.^8^ This diet induces classic metabolic syndrome symptoms such as insulin resistance,^9^ hyperglycemia, hyperinsulinemia, dyslipidemia, and pancreatic steatosis.^8,10^ We hypothesized that HFSC-induced pancreatic changes lead to proteome-level reprogramming inside pancreatic islets, impacting key pathways for β-cell survival and function.

To investigate this, we performed quantitative proteome profiling on isolated islets to identify molecular markers that contribute to early islet dysfunction in diet-induced metabolic syndrome. This approach aims at finding important proteins and pathways that may act as early indicators of β-cell stress and perhaps promote pancreatic dysfunction during metabolic syndrome progression.

## 2 Materials and Methods

### 2.1 Induction of Metabolic Syndrome

Healthy male C57BL/6J mice were kept under monitored conditions (25 ± 2 °C, 12-h light/dark cycle) following the recommendations of the Committee for Control and Supervision of Experiments on Animals, India. The studies were approved by the Institutional Animal Ethics Committee of St. Aloysius College (Deemed to be University), Mangaluru, India (Sanction No: SAC/IAEC/05/2019, Jan 28, 2019). One-month-old healthy male C57BL/6J mice weighing 18–20 g were randomly assigned to control and HFSC groups (*n* = 3). The control group was fed a standard diet, while the HFSC group received a specially prepared HFSC diet. Both groups were fed 5 g/day of their respective diets, as per Indian National Science Academy standards (2000), with unlimited access to water.^8,9^ These conditions were maintained for 150 days. The HFSC and control diets used in this study have been previously described and validated.^8–10^ Detailed characterization of this model, including dietary composition, body weight progression, and metabolic parameters, has been reported in these studies.

### 2.2 Preparation of Pancreatic Islets

The pancreas was dissected from the experimental group, and the splenic region was separated and washed with ice-cold Hank’s balanced salt solution (HBSS) under aseptic conditions. The pancreas was maintained in a bicarbonate-buffered medium at physiological pH, with excess fat tissues manually removed. Collagenase type IV (Sigma-Aldrich, St. Louis, MO) was injected at a concentration of 1 mg/mL in HBSS into the dissected pancreas repeatedly to disrupt the connective tissue.^11^ The finely chopped pancreatic tissues were further incubated with collagenase in a sterile glass tube at 37 ± 2 °C with vigorous shaking for 15 min. After digestion, the suspension was filtered to separate cell debris and fat, followed by washing with buffer and centrifugation at 500*g* for 1 min at room temperature.^11^ The islet suspension was then transferred to a sterile Petri dish. Pancreatic islets, appearing as yellow-sphere-like structures, were collected using sterile Pasteur pipettes under aseptic conditions while being observed through a dissection microscope.^10^

### 2.3 Isolation of Proteins from Pancreatic Islets

Approximately 150–200 pancreatic islets per sample were pooled and subjected to two rounds of washing with ice-cold phosphate-buffered saline (PBS), followed by centrifugation at 200*g* for 3 min for protein extraction. The resulting pellet was carefully transferred to a 1.5 mL safe-lock tube. After a final wash, the pellet was vigorously spun down in a microcentrifuge and aspirated to remove the remaining liquid. Subsequently, 50 μL of Rapigest extraction buffer (Waters, Manchester, U.K.) supplemented with protease inhibitors was added to the pellet, and the mixture was vigorously shaken. The reaction proceeded at room temperature for 10 min. Freeze–thaw cycles were performed twice by freezing the samples in liquid nitrogen for 1 min, followed by thawing at room temperature for 5 min. The resulting cell suspension was subjected to centrifugation at 21,000*g* for 30 min at 4 °C to obtain the supernatant containing protein, which was collected in a fresh tube. The protein samples were desalted and buffer-exchanged using Amicon Ultra 0.5 mL centrifugal filters (3 kDa molecular weight cutoff, Merck Millipore, Billerica, MA) with 50 mM ammonium bicarbonate buffer to remove salts and detergent prior to liquid chromatography-tandem mass spectrometry (LC-MS/MS) analysis as described by Aneesh et al. Following buffer exchange, the protein samples were quantified using the Pierce bicinchoninic acid kit (Thermo Fisher Scientific, Waltham, MA) according to the manufacturer’s protocol and subsequently diluted to a uniform concentration of 1 mg/mL. The protein samples from both HFSC and control groups were reduced using 100 mM 1,4-dithiothreitol (Sigma-Aldrich), followed by alkylation with 200 mM iodoacetamide (Sigma-Aldrich). Subsequently, overnight digestion was carried out using MS-grade trypsin (Sigma-Aldrich) at an enzyme-to-protein ratio of 1:25 (1 μg trypsin per 25 μg protein) at 37 °C.^12^

### 2.4 Protein Profiling Using ESI-nanoLC-MS/MS

Liquid chromatography analyses were performed using a nanoACQUITY UPLC system (Waters, Manchester, U.K.) coupled to a Synapt G2 High Definition MS (HDMSE) system (Waters). Data acquisition was controlled using MassLynx 4.1 SCN781 software. A binary solvent system consisting of solvent A (0.1% formic acid in water) and solvent B (0.1% formic acid in acetonitrile) was used. Peptides were initially trapped on a Symmetry C18 column (180 μm × 20 mm, 5 μm, Waters) and subsequently separated on an HSS T3 C18 analytical column (75 μm × 200 mm, 1.8 μm, Waters). The flow rate was maintained at 300 nL/min, and the column temperature was set at 35 °C. The autosampler temperature was maintained at 4 °C. Each biological sample was analyzed in technical duplicate (two LC-MS injections per sample), with an injection volume of 0.5 μL. The total LC gradient run time was 90 min. Mass spectrometry analysis was performed in positive electrospray ionization (ESI) mode with ion mobility separation (IMS) enabled. Calibration was performed using sodium iodide, and online mass correction was applied using leucine enkephalin (*m*/*z* 556.2766). The nano-ESI capillary voltage was set to 3.4 kV, with sample and extraction cone voltages of 40 and 4 V, respectively. Nitrogen was used as the IMS gas at 90 mL/min. Collision energy was ramped from 20 to 45 eV. Data was acquired in resolution mode and recorded in continuum format.^12^

### 2.5 Proteomic Data Analysis and Statistical Processing

Raw mass spectrometry data were processed using Progenesis QI for Proteomics v4.2 (Nonlinear Dynamics, Newcastle upon Tyne, U.K.). Peptide identification was performed against the UniProt *Mus musculus* reference proteome database (downloaded 17 Jan, 2023), with the protein-level false discovery rate (FDR) controlled at 1%. The database included both reviewed and unreviewed protein sequences, and both canonical proteins and isoforms were considered during the database search. Search parameters included one missed cleavage for trypsin specificity, fixed carbamidomethylation of cysteine, and variable oxidation of methionine.^12^ For quantitative analysis, normalized protein abundance values were obtained within Progenesis QI. Proteins were retained for downstream analysis if they satisfied the following criteria: peptide count ≥ 2, and unique peptide ≥ 1. Differential expression analysis was performed using one-way analysis of variance (ANOVA) implemented within Progenesis QI. Multiple testing correction was applied using the Benjamini–Hochberg false discovery rate (FDR) method, and adjusted *p*-values (*q*-values) were calculated. Proteins were considered significantly differentially expressed if they met the following criteria: i.e., adjusted *p*-value (*q*-value) ≤ 0.05, and a fold change ≥ 1.5. Technical duplicates were averaged prior to statistical analysis, and all statistical comparisons were performed using biological replicates (*n* = 3 per group).^12^

### 2.6 Bioinformatics Analysis and Visualization of Differentially Expressed Proteins (DEPs)

Normalized protein abundance values exported from Progenesis QI for Proteomics were used for downstream bioinformatics analyses. Multivariate statistical analyses, including principal component analysis (PCA) and Pearson correlation analysis, were performed using MetaboAnalyst 6.0 to evaluate global variation and reproducibility among biological replicates. Pearson correlation matrices were generated to assess similarity between samples based on protein abundance profiles. In addition, hierarchical clustering heatmaps were generated to visualize the expression patterns of differentially expressed proteins (DEPs) across experimental groups. Clustering was performed for both proteins and samples using Euclidean distance and Ward’s linkage method following autoscaling (*z*-score transformation) of features.^12^ Volcano plots were generated using the SRPlot online platform (https://www.bioinformatics.com.cn) to visualize the distribution of significantly upregulated and downregulated proteins.

### 2.7 Gene Ontology (GO) and KEGG Enrichment Analysis of DEPs

Gene ontology (GO) and Kyoto encyclopedia of genes and genomes (KEGG) pathway enrichment analyses were performed using the web-based functional annotation tool ShinyGO (http://bioinformatics.sdstate.edu/go/). ShinyGO is an R-based platform that integrates multiple biological databases for the functional enrichment analysis and visualization. To better understand the functional differences associated with protein regulation, enrichment analyses were performed separately for the upregulated and downregulated DEPs. GO enrichment results were categorized into biological process (BP), molecular function (MF), and cellular component (CC). KEGG pathway analysis was conducted to identify significantly enriched metabolic and signaling pathways associated with DEPs. Statistically significant GO terms and KEGG pathways were identified using an adjusted *p*-value (FDR) threshold of <0.05.

### 2.8 Protein–Protein Interaction (PPI) Network Analysis

The search tool for retrieval of interacting genes (STRING) (https://string-db.org) database, which integrates both known and predicted PPIs, can be applied to predict functional interactions of proteins. To seek potential interactions between DEPs, the STRING tool was employed. Active interaction sources, including text mining, experiments, databases, and coexpression, as well as species limited to “*M. musculus*” and an interaction score >0.4, were applied to construct the PPI networks.

## 3 Results

### 3.1 Proteome Identification and Differential Expression Analysis

To investigate the molecular changes associated with HFSC-induced pancreatic alterations, quantitative proteome profiling of isolated pancreatic islets was performed using ESI-nanoLC-MS/MS. Label-free quantitative proteomic analysis identified a total of 386 proteins across control and HFSC diet-fed groups. To ensure high-confidence protein identification, only proteins with a peptide count ≥ 2 and at least one unique peptide were retained for downstream analysis. Differential expression analysis was performed using one-way ANOVA followed by FDR correction. Proteins with *q* ≤ 0.05 and log_2_ fold change ≥ 0.585 (corresponding to fold change ≥ 1.5) were considered significantly DEPs.

On the basis of these criteria, 30 DEPs were identified, including 19 upregulated and 11 downregulated proteins in HFSC islets compared with controls (Figure 1). Upregulated proteins included INS1, PAH, GATD3, DMDTt1, HNRNPA3, RPS7, RPS13, ACTB, SYNCRIP, HSPA1L, SDF2L1, RPS, COPG1, RRBP1, and EEF1A1 among others. Downregulated proteins included AKR1B1, HSP90B1, HSP90AB1, EIF4A2, PFN1, PNLIPRP2, CCDC88C, MANF, GFUS, and SH3GL3.

**Figure 1:**
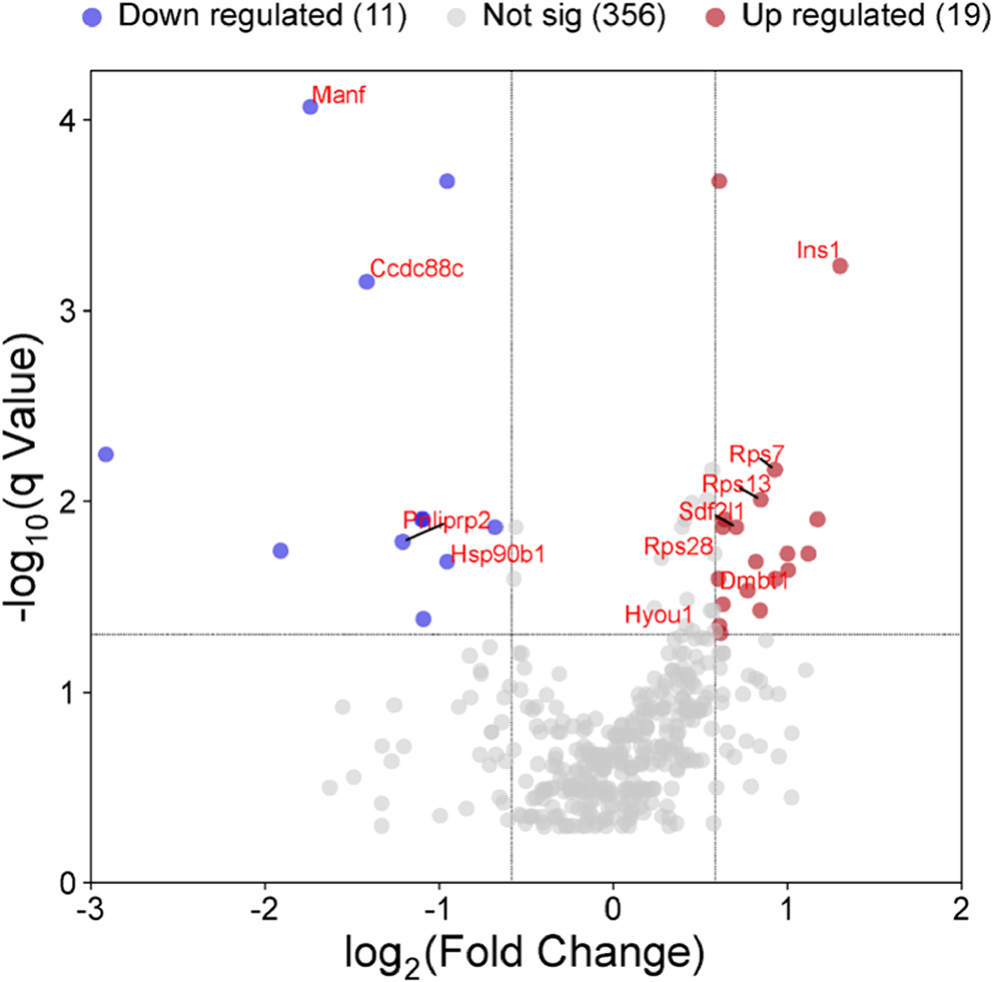
Volcano plot showing differentially expressed proteins in pancreatic islets from HFSC-fed mice compared to controls. The *x*-axis represents the fold change (log_2_ FC), while the *y*-axis represents each protein’s statistical significance (−log_10_ *q*-value). Proteins were filtered using a peptide count of ≥2 and at least one unique peptide. Differential expression was defined as *q* ≤ 0.05 and log_2_ fold change ≥ 0.585. Red dots indicate upregulated proteins and blue dots indicate downregulated proteins.

A complete list of all identified proteins, along with their normalized abundance values across biological replicates, is provided in Table S1 (Supporting Information). The list of significantly differentially expressed proteins, including the associated statistical parameters and normalized abundance values used for downstream analyses, is provided in Table S2 (Supporting Information).

### 3.2 Multivariate and Correlation Analysis

To evaluate global proteomic variation and sample clustering, principal component analysis (PCA) was performed using normalized abundance values of all 386 quantified proteins exported from Progenesis QI without additional transformation or scaling. The first two principal components explained 85.6% of the total variance (PC1, 64.7%, PC2, 20.9%). Clear separation between control and HFSC groups was observed primarily along PC1, indicating distinct proteomic profiles between the two experimental conditions. No extreme outliers were detected, and all biological replicates clustered according to their respective groups (Figure 2A).

**Figure 2:**
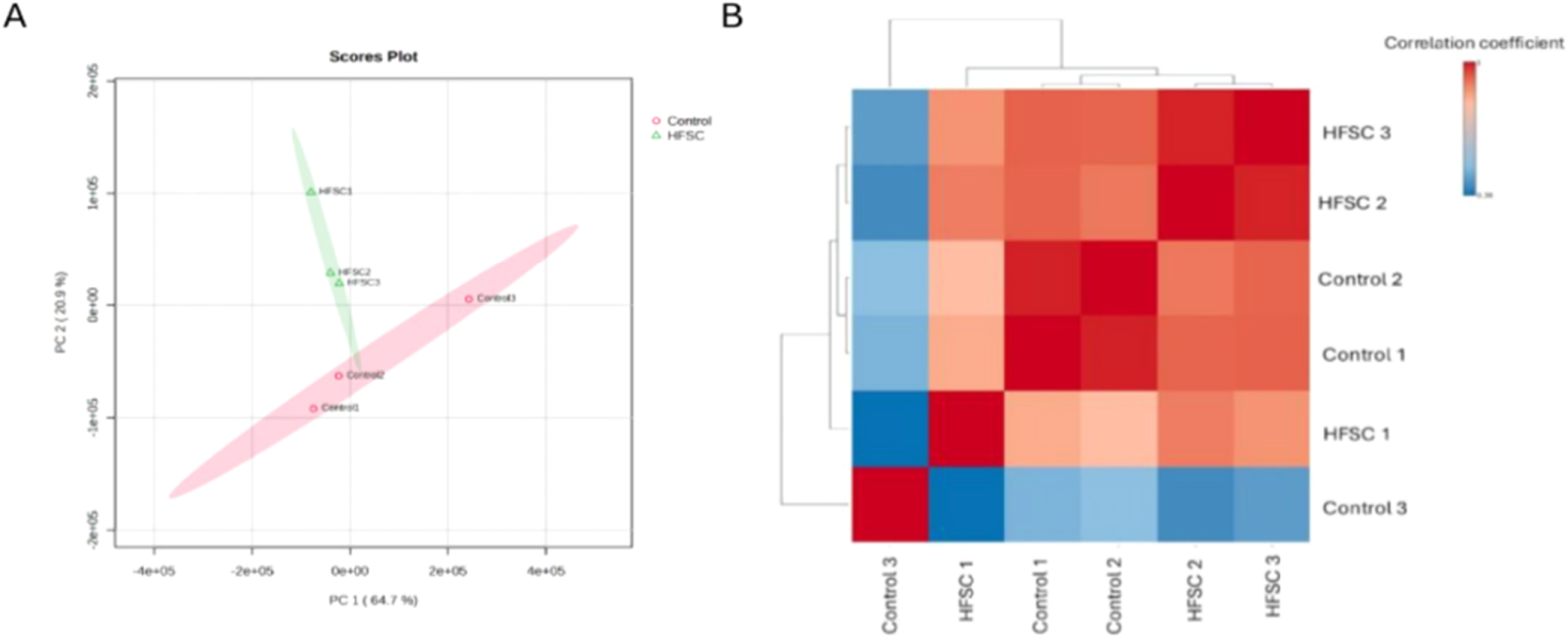
Multivariate analysis and correlation assessment of proteomic samples. (A) Principal component analysis (PCA) score plot generated from normalized protein abundance values showing clear separation between control and HFSC-fed samples. The first two principal components explain a large proportion of the total variance (PC1: 64.7%, PC2: 20.9%), indicating distinct proteomic profiles between experimental groups. (B) Pearson correlation heatmap illustrating pairwise correlation coefficients among biological replicates. Darker colors indicate higher correlation, whereas lighter colors indicate lower correlation. Samples cluster primarily according to dietary groups, with strong intragroup correlation among HFSC and control replicates, demonstrating high reproducibility and consistent proteomic signatures within each experimental condition.

To further assess reproducibility among biological replicates, Pearson correlation analysis was performed across all quantified proteins. High intragroup correlation coefficients were observed within both control and HFSC groups, whereas intergroup correlations were comparatively lower. Hierarchical clustering based on Pearson correlation further segregated samples according to their respective experimental groups (Figure 2B). Within the control group, Control1 and Control2 showed very high correlation (*r* = 0.987), whereas Control3 exhibited comparatively lower correlation with the other controls (*r* ≈ 0.49–0.52), indicating moderate biological variability. In contrast, HFSC replicates demonstrated consistently high intragroup correlation (*r* = 0.87–0.99). No extreme outliers were detected; therefore, all samples were retained for downstream analysis.

Hierarchical clustering heatmap analysis was performed using normalized abundance values with feature autoscaling (*z*-score transformation), Euclidean distance, and Ward’s linkage method. Both proteins and samples were clustered, and the resulting heatmap showed clear segregation between control and HFSC groups based on the expression patterns of differentially expressed proteins (Figure 3). The coefficient of variation (CV) across quantified proteins was calculated to assess reproducibility among biological replicates. The median CV was 41.2% for control samples and 21.3% for HFSC samples, indicating acceptable reproducibility of protein abundance measurements (Supporting Figure S1).

**Figure 3:**
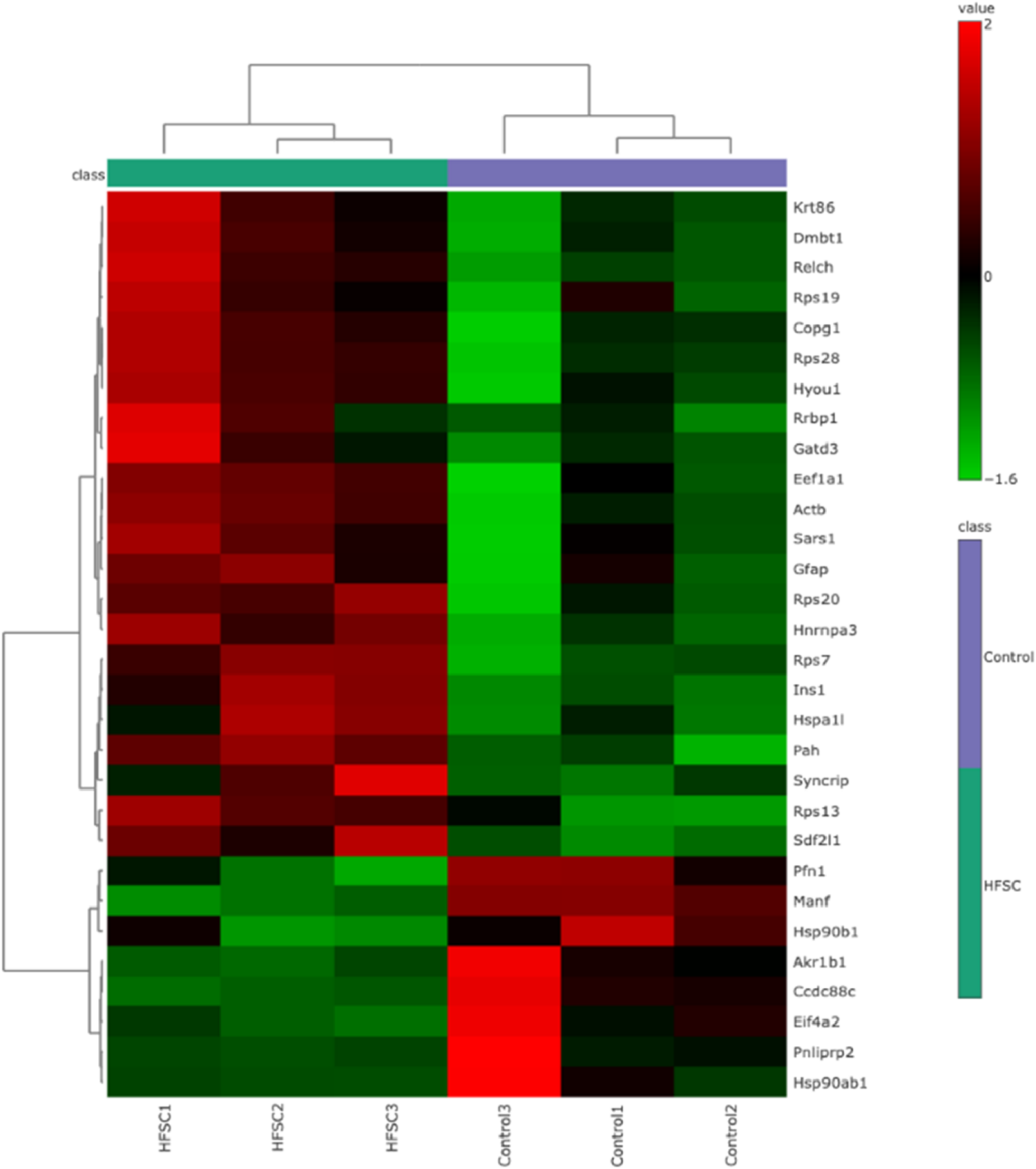
Hierarchical clustering heatmap of differentially expressed proteins in pancreatic islets from control and HFSC-fed mice. Normalized protein abundance values were autoscaled (*z*-score transformation) prior to hierarchical clustering. Rows represent differentially expressed proteins and columns represent biological replicates from control and HFSC-fed groups. Clustering of both proteins and samples was performed using Euclidean distance and Ward’s linkage method. The color scale represents relative protein abundance, with red indicating higher expression and green indicating lower expression across samples. The dendrogram shows clear segregation of HFSC-fed and control samples, indicating distinct proteomic signatures associated with HFSC-induced metabolic alterations in pancreatic islets.

### 3.3 Functional Enrichment Analysis of Differentially Expressed Proteins

To elucidate the biological processes associated with HFSC-induced proteomic alterations in pancreatic islets, GO and KEGG pathway enrichment analyses were performed separately for upregulated and downregulated proteins. Upregulated proteins demonstrated coordinated enrichment of translational and proteostasis-related pathways. GO biological process analysis revealed significant enrichment in cytoplasmic translation, ribosomal small-subunit biogenesis, de novo post-translational protein folding, chaperone cofactor-dependent protein refolding, and positive regulation of signal transduction by p53 class mediator (Figure 4A). Consistently, GO molecular function analysis showed enrichment of structural constituents of ribosome, mRNA binding activities (including 5′-UTR and 3′-UTR binding), adenosine triphosphate (ATP)-dependent protein folding chaperone activity, and ubiquitin-protein transferase regulator activity (Figure 4B). GO cellular component analysis further indicated overrepresentation of cytosolic ribosomes, small ribosomal subunits, preribosomal complexes, small-subunit processome structures, and endoplasmic reticulum (ER) chaperone complexes, including ER lumen components (Figure 4C). KEGG pathway enrichment analysis supported these findings, highlighting significant enrichment of ribosome and protein processing in endoplasmic reticulum pathways, indicating increased translational activity and enhanced ER proteostasis demands in HFSC-fed islets (Figure 4A). Additional enrichment of the longevity-regulating pathway, across multiple species, was also observed, reflecting shared components involved in cellular stress responses and metabolic regulation (Figure 4D).

**Figure 4:**
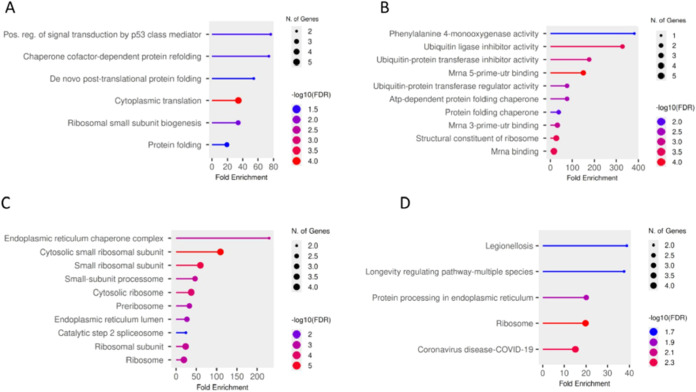
Functional enrichment analysis of upregulated proteins. GO and KEGG pathway enrichment analysis of upregulated proteins in HFSC-fed pancreatic islets. Enriched categories include (A) biological process, (B) molecular function, (C) cellular component, and (D) KEGG pathways. The size of the dots represents the number of genes associated with each function, while the color intensity indicates the statistical significance (−log_10_ FDR), with darker colors representing higher significance.

In contrast, downregulated proteins were predominantly enriched in metabolic and redox-associated pathways. GO biological process analysis identified enrichment in hexitol metabolic process, galactolipid metabolic and catabolic processes, GDP-mannose metabolic process, alditol biosynthetic process, and l-ascorbic acid biosynthesis (Figure 5A). GO molecular function enrichment showed overrepresentation of oxidoreductase activity acting on the CH–OH group of donors, including NAD/NADP-dependent oxidoreductase activity, as well as ATP hydrolysis activity (Figure 5B). GO cellular component analysis highlighted enrichment of sarcoplasmic reticulum lumen and endoplasmic reticulum lumen (Figure 5C).

**Figure 5:**
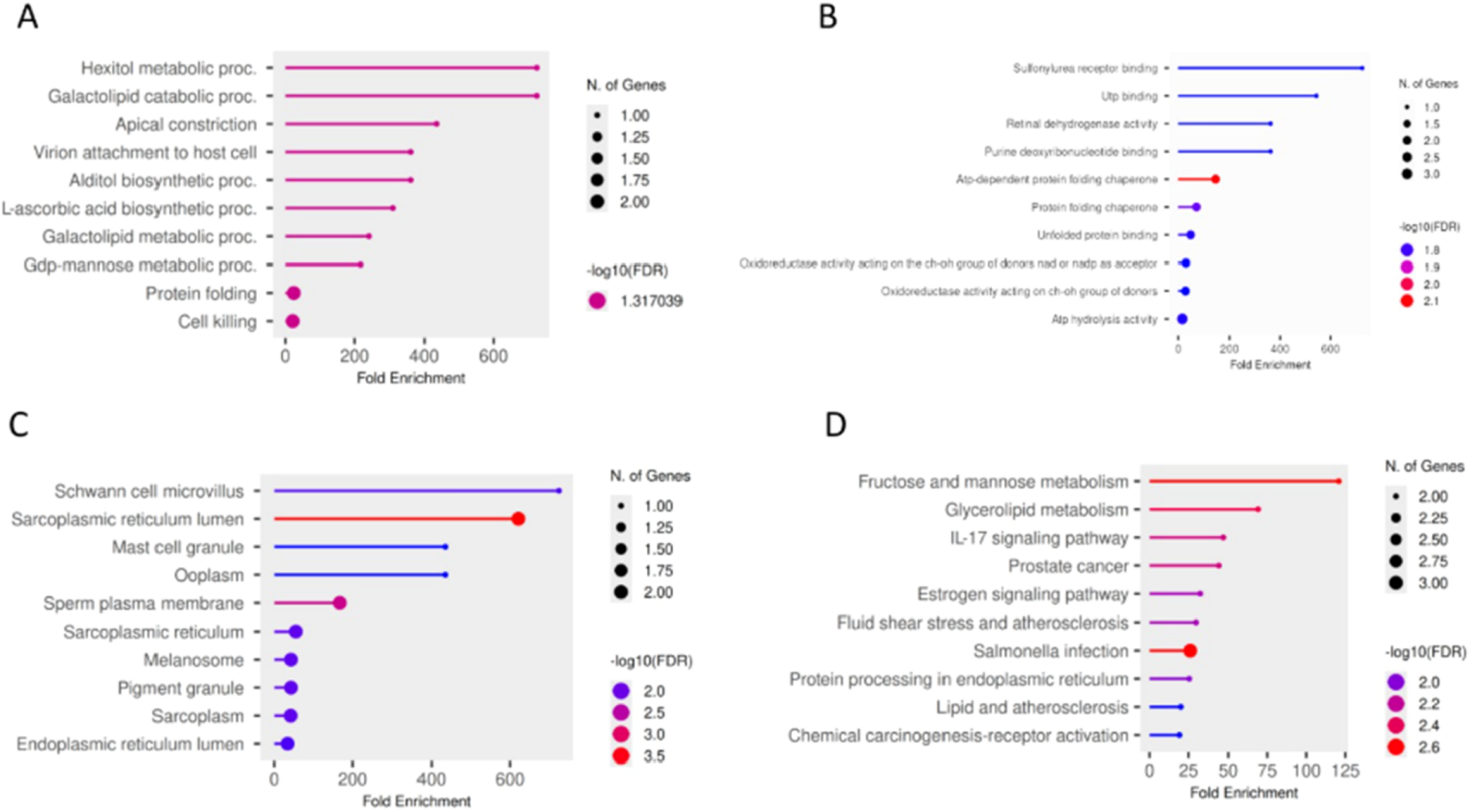
Functional enrichment analysis of downregulated proteins. GO and KEGG pathway enrichment analysis of downregulated proteins in HFSC-fed pancreatic islets. Enriched categories include (A) biological process, (B) molecular function, (C) cellular component, and (D) KEGG pathways, highlighting alterations in carbohydrate metabolism, oxidoreductase activity, and lipid metabolic pathways.

Consistent with these observations, KEGG pathway analysis revealed significant enrichment of fructose and mannose metabolism and glycerolipid metabolism pathways, suggesting alterations in carbohydrate and lipid metabolic processes in HFSC-fed islets (Figure 5D). Enrichment of signaling pathways such as IL-17 signaling and estrogen signaling likely reflects shared regulatory components associated with cellular stress and inflammatory responses. Together, these enrichment analyses indicate that HFSC feeding induces enhanced translational activity and ER proteostasis responses accompanied by suppression of metabolic pathways, suggesting substantial remodeling of protein synthesis, folding capacity, and metabolic homeostasis in pancreatic islets.

### 3.4 Protein–Protein Interaction Network Analysis

To explore potential functional relationships among the DEPs, a PPI network was constructed using the STRING database with an interaction score threshold of 0.4 and species restricted to *M. musculus*. The resulting network revealed several interconnected modules among the DEPs (Figure 6). Cluster analysis identified three major functional interaction groups. The first cluster consisted primarily of ER-associated chaperone proteins, including HSP90B1, HYOU1, SDF2L1, MANF, and HSPA1L, forming a densely connected interaction module. The second cluster was enriched with ribosomal- and translation-related proteins, including RPS7, RPS13, RPS20, RPS28, and EEF1A1, along with RNA-binding proteins such as SYNCRIP and HNRNPA3. A third cluster contained proteins associated with metabolic regulation, vesicle trafficking, and cytoskeletal organization, including ACTB, PFN1, AKR1B1, PAH, COPG1, RELCH, and PNLIPRP2.

**Figure 6:**
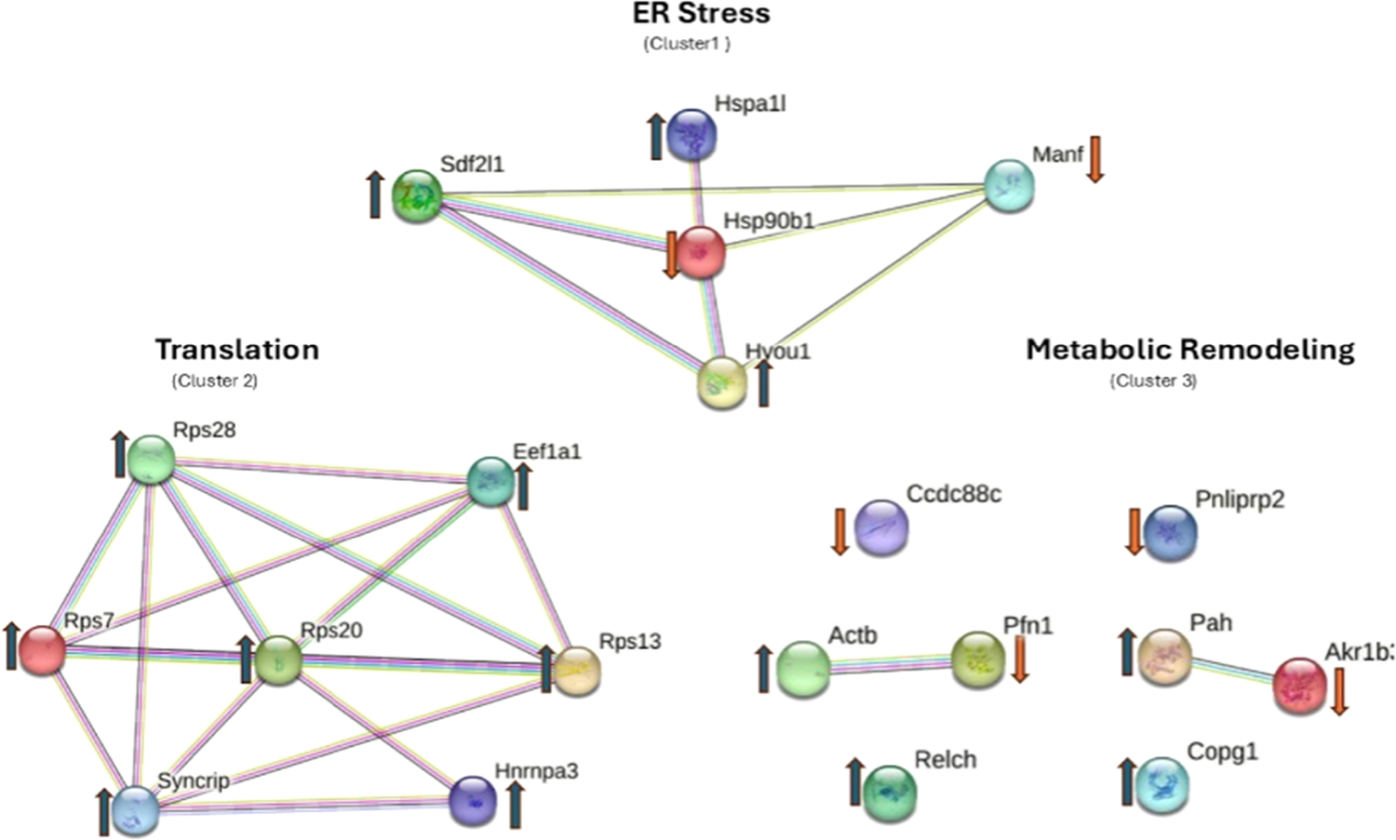
Protein–protein interaction network of differentially expressed proteins. The interaction network was generated using the STRING database with an interaction score threshold of 0.4 and species restricted to *M. musculus*. Three major functional clusters were identified. Cluster 1 represents ER stress and proteostasis-associated chaperone proteins, including HSP90B1, HYOU1, SDF2L1, and MANF. Cluster 2 contains ribosomal and translational machinery proteins such as RPS7, RPS13, RPS20, RPS28, and EEF1A1, indicating enhanced protein synthesis activity. Cluster 3 consists of proteins involved in metabolic regulation, vesicle trafficking, and cytoskeletal organization, suggesting metabolic and structural remodeling of pancreatic islet cells under HFSC-induced metabolic stress. Upward arrows (blue) indicate upregulated proteins, while downward arrows (orange) indicate downregulated proteins in HFSC-fed pancreatic islets.

Representative boxplots of selected differentially expressed proteins further supported the observed expression patterns across biological replicates. ER stress-associated proteins (HYOU1, MANF, SDF2L1) and the ribosomal protein RPS7 (Figure 7A–D) showed consistent differences in abundance between control and HFSC-fed islets, confirming the direction and reproducibility of differential expression identified in the proteomic analysis.

**Figure 7:**
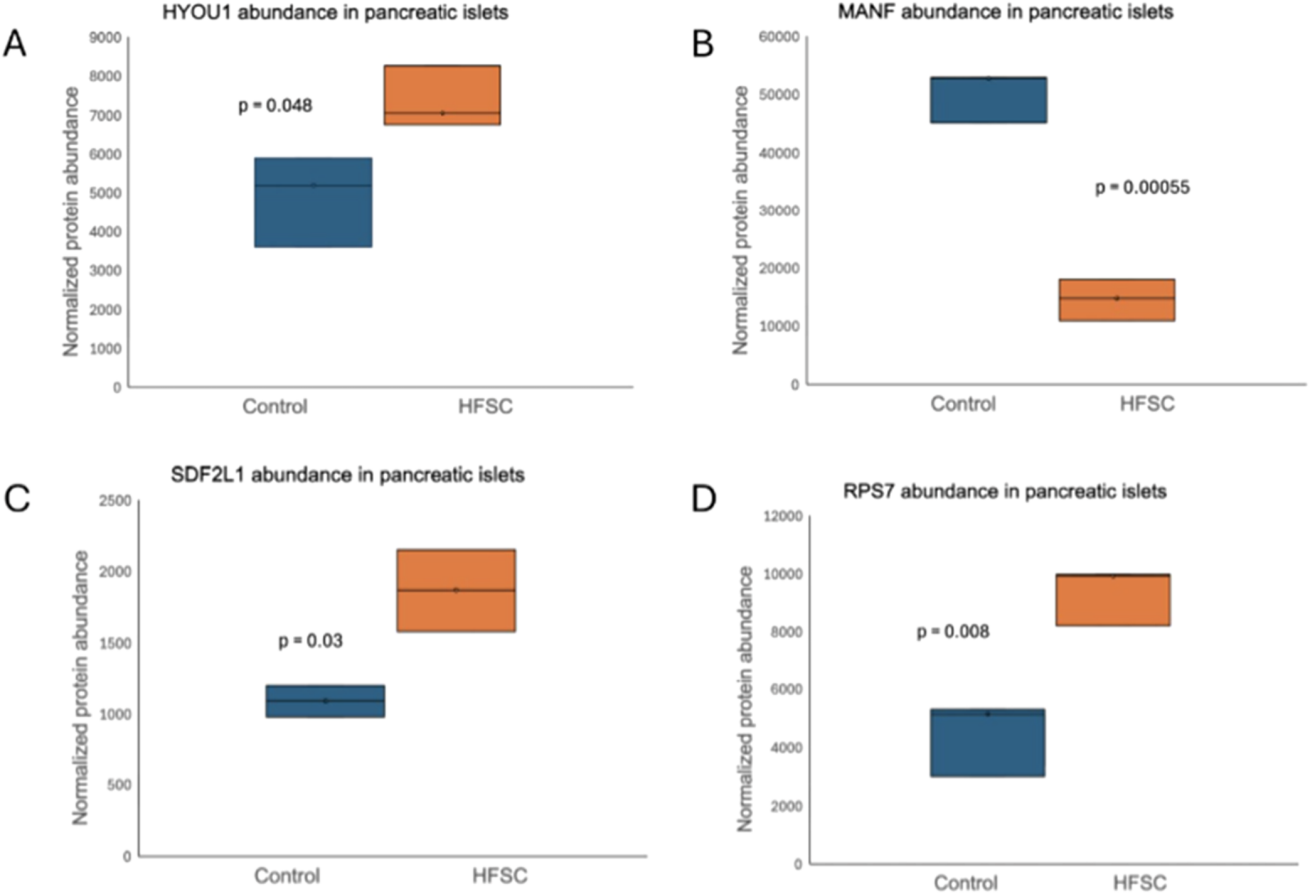
Boxplots of representative differentially expressed proteins. Boxplots showing normalized abundance of selected proteins identified by proteomic analysis in control and HFSC-fed mice pancreatic islets. ER stress-associated proteins: (A) HYOU1, (B) MANF, and (C) SDF2L1, together with the ribosomal protein (D) RPS7, demonstrate distinct expression patterns between experimental groups, supporting the differential expression identified in the proteomic analysis. Statistical significance was assessed using an unpaired two-tailed *t*-test.

## 4 Discussion

The present study demonstrates that long-term HFSC feeding induces significant molecular remodeling in pancreatic islets, highlighting pancreatic β-cells as key targets of diet-induced metabolic stress during metabolic syndrome. Previous histological observations from our laboratory showed that HFSC feeding leads to inflammatory infiltration, pancreatic steatosis, and islet hypertrophy in C57BL/6J mice, suggesting increased endocrine demand and altered exocrine–endocrine interactions.^8,10^ The present proteomic findings provide molecular insight into these structural alterations by revealing enhanced protein synthesis, increased chaperone activity, and activation of ER stress-associated pathways. The upregulation of major proteostasis regulators, including HSP90B1,^13,14^ HYOU1,^15^ SDF2L1,^16^ and MANF,^17^ suggests that β-cells enhance their protein folding capacity to cope with increased biosynthetic demand. Persistent activation of these stress-response networks may ultimately contribute to progressive β-cell dysfunction during the progression of metabolic syndrome.^18^

The structural and inflammatory alterations observed in the pancreas during HFSC feeding are consistent with the proteomic changes identified in isolated islets.^8^ Increased expression of proteins involved in ribosomal assembly, cytoplasmic translation, and protein folding indicates elevated biosynthetic demand to support enhanced insulin production during metabolic stress.^19^ However, sustained increases in insulin biosynthesis place a considerable burden on the ER, triggering activation of the unfolded protein response (UPR).^20^ Activation of ER stress signaling pathways and induction of chaperones such as HSP90B1, HYOU1, MANF, and SDF2L1 are well-established adaptive mechanisms that enhance protein folding capacity and maintain proteostasis.^13,15–17,21^ Nevertheless, prolonged ER stress may eventually lead to β-cell dysfunction, dedifferentiation, and apoptosis, which are key contributors to the development of type 2 diabetes.^22,23^ In addition to ER stress responses, the enrichment of oxidoreductase activity and redox-related metabolic processes suggests that metabolic overload may disrupt cellular redox homeostasis in pancreatic islets. Proteins associated with redox regulation and mitochondrial function, including PRDX1 ^24^ and VCP,^25^ were also observed in the data set, suggesting potential alterations in cellular redox balance during sustained insulin production. The combined effects of ER stress and redox imbalance are known to compromise β-cell survival and impair insulin secretion, thereby contributing to metabolic deterioration during chronic nutrient excess.^26^

The downregulation of proteins associated with carbohydrate and lipid metabolic processes, including AKR1B1, PNLIPRP2, and GFUS, suggests early alterations in metabolic regulation within pancreatic islets of HFSC-fed mice. In healthy β-cells, the ability to efficiently utilize multiple nutrient sources, including glucose, fatty acids, and amino acids, is essential for maintaining appropriate insulin secretion in response to nutrient availability. The reduced abundance of enzymes involved in these metabolic pathways may therefore reflect impaired metabolic adaptability under chronic HFSC exposure. Notably, several of these proteins, including AKR1B1 and GFUS, are associated with oxidoreductase activity,^27,28^ suggesting potential alterations in cellular redox balance within the pancreatic islet microenvironment. Pancreatic β-cells possess relatively limited antioxidant defense capacity compared with other tissues, rendering them particularly vulnerable to oxidative stress.^29^ Consequently, reduced expression of redox-regulating proteins may weaken the ability of β-cells to counteract oxidative challenges during chronic nutrient excess. Disruption of redox homeostasis has been proposed to contribute to β-cell dysfunction by promoting oxidative damage and exacerbating ER stress, ultimately impairing protein folding and insulin secretion.^30^ Together, these observations suggest a transition from adaptive metabolic responses toward early stress-associated remodeling of pancreatic islets during prolonged HFSC exposure.

The proteomic alterations observed in the present study are broadly consistent with previously reported molecular responses of pancreatic islets exposed to metabolic stress.^26,31^ Earlier transcriptomic and proteomic investigations in obesity, insulin resistance, and type 2 diabetes models have similarly reported increased ribosomal protein expression, activation of translational machinery,^32^ and induction of ER stress pathways in pancreatic β-cells.^33,34^ These responses are generally interpreted as adaptive mechanisms that support increased insulin biosynthesis under conditions of metabolic overload. Similarly, oxidative stress-related pathways have frequently been reported in islets exposed to chronic metabolic stress, reflecting elevated mitochondrial activity and reactive oxygen species production during sustained insulin secretion.^35^ Therefore, the enrichment of ribosomal proteins and ER-associated chaperone pathways observed in the present data set aligns with previously described molecular adaptations of pancreatic islets undergoing metabolic stress.^36,37^

To assess the biological significance of our data set, we compared our proteomic profile with findings from prior pancreatic islet proteomics studies. In the present HFSC-fed C57BL/6J mouse model, we observed upregulation of ribosomal proteins and ER-associated chaperones such as HSP90B1 and HYOU1, together with downregulation of several metabolic enzymes and oxidoreductases. Similar molecular patterns have been reported in earlier studies investigating pancreatic islet responses to metabolic stress.^38–40^ For example, Chan et al. reported increased abundance of chaperone proteins and insulin secretion-associated machinery in isolated mouse islets, suggesting activation of unfolded protein response pathways.^41^ Likewise, Nakashima et al. observed enrichment of ER stress-related proteins and secretory pathway components in porcine islets, accompanied by reduced abundance of enzymes involved in lipid metabolism.

To further contextualize the present findings, it is important to consider the overall proteome coverage obtained in this study. A total of 386 proteins were identified in collagenase-isolated pancreatic islets using label-free LC-MS/MS analysis on the Synapt G2 HDMSE platform. Although this number is lower than that reported in large-scale whole-tissue proteomics data sets generated using high-resolution Orbitrap mass spectrometry, several biological and technical factors likely contribute to the observed proteome depth.^42,43^ Pancreatic islets represent a small endocrine compartment that constitutes only a minor fraction of the total pancreatic mass, and collagenase-based isolation yields limited protein quantities.^37,44^ Consequently, detection of low-abundance regulatory proteins may be constrained in islet-specific proteomic analyses. Furthermore, while the Synapt G2 HDMSE platform provides robust label-free quantification and reproducible data acquisition, its analytical depth is generally lower than that of newer high-resolution mass spectrometry platforms.^45,46^ Nevertheless, the consistent enrichment of translational, ER stress, and proteostasis-associated pathways across biological replicates indicates that the achieved proteome depth was sufficient to capture key molecular adaptations associated with HFSC-induced metabolic stress in pancreatic islets.

The direction-specific enrichment analyses further revealed coordinated proteomic reprogramming of pancreatic islets under chronic HFSC exposure. Upregulated proteins were predominantly enriched in pathways associated with ribosomal biogenesis, cytoplasmic translation, and ER-associated chaperone complexes, indicating increased biosynthetic demand and activation of proteostasis networks.^26,47^ This pattern is consistent with compensatory enhancement of protein synthesis and folding capacity in response to sustained metabolic overload.^48^ In contrast, downregulated proteins were enriched in carbohydrate and lipid metabolic processes^40,49^ as well as oxidoreductase activities,^50^ suggesting reduced metabolic flexibility and altered redox balance within islet cells. The simultaneous activation of translational and ER stress-related pathways alongside suppression of metabolic pathways suggests a shift from metabolic homeostasis toward stress-adaptive protein synthesis in pancreatic islets.

The differential expression of proteins associated with cellular stress responses and proteostasis raises the possibility that prolonged metabolic stress may create a permissive cellular environment for pathological remodeling. Although epidemiological studies have linked high-fat dietary patterns with an increased risk of pancreatic cancer,^51,52^ the present proteomic data set does not directly assess oncogenic transformation or tumorigenic markers. Therefore, any potential link between HFSC-induced proteomic alterations and malignant progression remains speculative and requires further mechanistic investigation. The present findings primarily highlight stress-associated molecular reprogramming characterized by enhanced translational activity, ER stress signaling, and oxidative imbalance, which represent well-recognized adaptive responses of pancreatic β-cells to metabolic overload. In line with these observations, the schematic model presented in Figure 8 summarizes the proposed mechanism whereby HFSC-induced metabolic overload increases insulin biosynthesis, leading to ER stress activation, oxidative stress generation, and alterations in proteostasis within pancreatic β-cells, as suggested by the proteomic data set.

**Figure 8:**
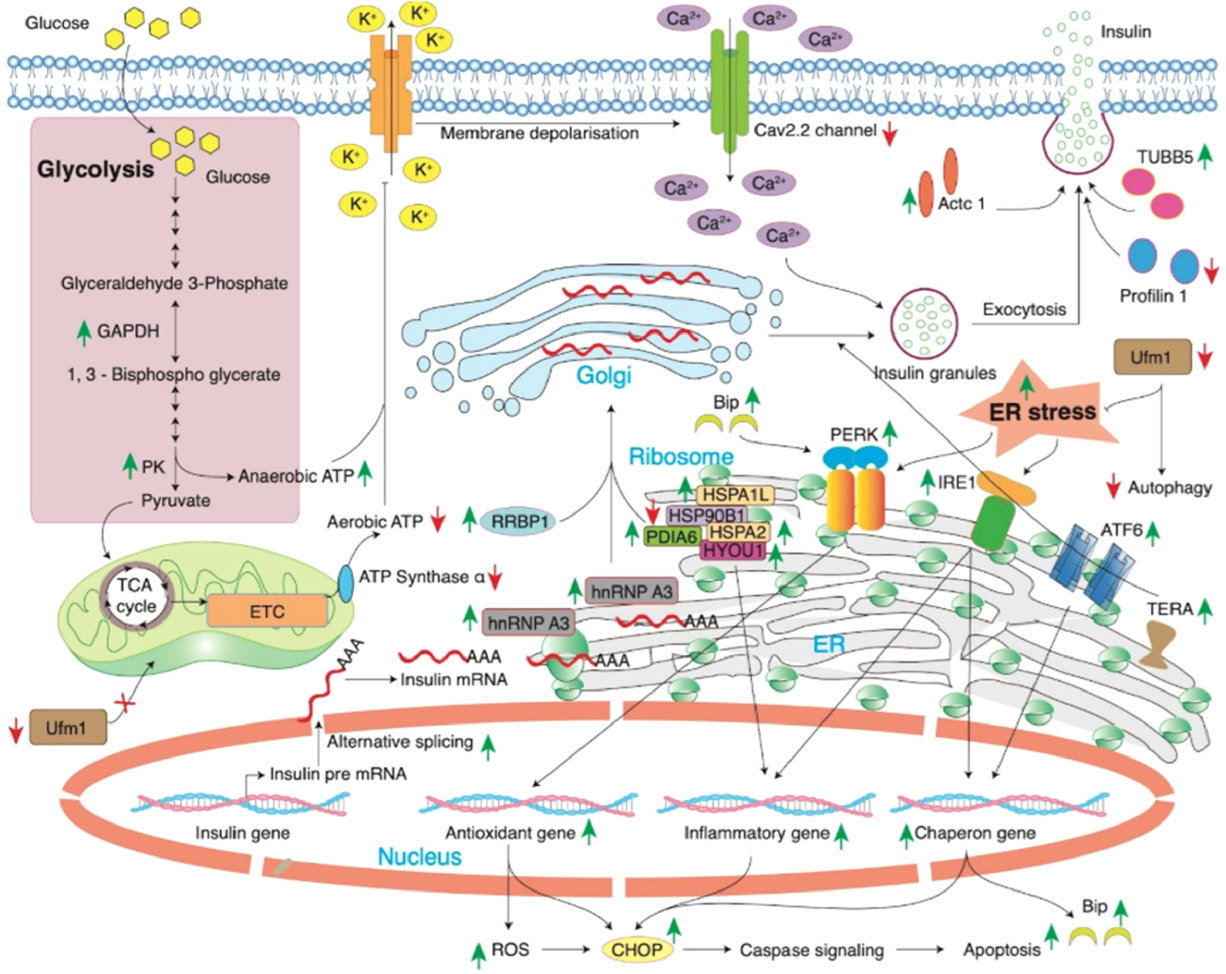
Proposed schematic model illustrating HFSC-induced metabolic and proteostasis stress in pancreatic islets. A schematic representation summarizing the molecular mechanisms associated with HFSC-induced metabolic stress in pancreatic islets based on the present proteomic data set. The model integrates proteins identified in the data set together with known components of β-cell stress-response pathways. Differentially expressed proteins meeting the statistical criteria are highlighted based on the proteomic analysis, whereas other proteins shown in the schematic exhibited directional expression changes in the data set but did not meet the stringent statistical thresholds applied for differential expression. Upregulation of ER-associated chaperones, including HSP90B1, HYOU1, SDF2L1, and MANF, suggests activation of unfolded protein response pathways in response to increased biosynthetic demand. Alterations in translational machinery, metabolic enzymes, and cytoskeletal proteins may collectively influence insulin biosynthesis, vesicle trafficking, and cellular stress responses under chronic HFSC exposure.

The present study has several limitations that should be considered in interpreting the results. The overall proteome depth was modest compared with large-scale whole-tissue proteomic data sets, likely reflecting the limited protein yield obtained from collagenase-isolated pancreatic islets and the analytical depth of the Synapt G2 HDMSE platform used in this study. Consequently, low-abundance regulatory proteins may not have been comprehensively detected.

Also, the number of biological replicates was limited, which is a common constraint in discovery-based proteomic studies involving small and technically challenging samples, such as pancreatic islets. Although multivariate analyses, including principal component analysis, Pearson correlation, hierarchical clustering, and coefficient of variation assessment, demonstrated clear group separation and acceptable reproducibility, increasing the sample size in future studies would further strengthen statistical power and generalizability.

Although the proteomic analysis provides valuable insights into molecular pathways altered under HFSC-dietary conditions, functional validation of selected candidate proteins using orthogonal experimental approaches such as immunoblotting or targeted proteomics was not performed. Future studies incorporating deeper proteomic coverage, larger cohorts, and experimental validation will therefore be important to further elucidate the mechanistic roles of these proteins in diet-induced pancreatic-islet dysfunction.

## 5 Conclusion

In summary, the present study provides proteomic insights into molecular alterations occurring in pancreatic islets under chronic HFSC-dietary exposure. Proteomic profiling identified 30 DEPs in pancreatic islets of HFSC-fed mice compared to controls. The differential expression of proteins involved in the translational machinery, ER protein processing, and metabolic pathways suggests that pancreatic islets undergo molecular adaptations to diet-induced metabolic stress. Enrichment of pathways related to ribosomal biogenesis, cytoplasmic translation, and ER-associated proteostasis indicates an increased biosynthetic demand and activation of cellular stress-response mechanisms. Conversely, the downregulation of proteins associated with carbohydrate and lipid metabolism suggests an altered metabolic homeostasis within islet cells.

These findings suggest that pancreatic islets undergo early adaptive molecular responses to metabolic stress that may occur prior to overt metabolic dysfunction. The observed proteomic alterations provide insight into how diet-induced metabolic syndrome may influence islet proteostasis and stress-response pathways, potentially contributing to β-cell vulnerability under chronic metabolic stress. Collectively, these results provide supporting proteomic evidence of molecular remodeling in pancreatic islets during HFSC-induced metabolic stress and highlight pathways potentially involved in early islet dysfunction associated with metabolic syndrome. Further studies integrating functional validation and deeper proteomic coverage will be important to clarify the mechanistic roles of these proteins in diet-induced pancreatic pathology.

## Supplementary Material





## Data Availability

The mass spectrometry proteomics data have been deposited to the ProteomeXchange Consortium via the PRIDE partner repository with the data set identifier PXD057058 and 10.6019/PXD057058 (https://www.ebi.ac.uk/pride/archive/projects/PXD057058). All of the data related to this study have been included in the supporting files.

## Notes

Supporting figure (S1 CV distribution plot) (PDF)
Supporting Tables (S1-complete proteome data set with normalized abundance values) and (S2-differentially expressed proteins and associated statistical parameters) (XLSX)

## References

[1] Bovolini A., Garcia J., Andrade M. A., Duarte J. A. (2021). Metabolic Syndrome
Pathophysiology and Predisposing Factors. Int.
J. Sports Med..

[2] Aslanoglou D., Bertera S., Sánchez-Soto M. (2021). Dopamine regulates pancreatic
glucagon and insulin secretion via adrenergic and dopaminergic receptors. Transl. Psychiatry.

[3] Chan T. T., Tse Y. K., Lui R. N. S. (2022). Fatty Pancreas Is Independently
Associated With Subsequent Diabetes Mellitus Development: A 10-Year
Prospective Cohort Study. Clin. Gastroenterol.
Hepatol..

[4] Gao M., Ma Y., Liu D. (2015). High-Fat Diet-Induced Adiposity, Adipose Inflammation,
Hepatic Steatosis and Hyperinsulinemia in Outbred CD-1 Mice. PLoS One.

[5] Möller K., Jenssen C., Braden B. (2023). Pancreatic changes with
lifestyle and age: What is normal and what is concerning?. Endosc. Ultrasound.

[6] Diamanti K., Cavalli M., Pereira M. J. (2022). Organ-specific metabolic
pathways distinguish prediabetes, type 2 diabetes, and normal tissues. Cell Rep. Med..

[7] Nyalwidhe J. O., Grzesik W. J., Burch T. C. (2017). Comparative quantitative
proteomic analysis of disease stratified laser captured microdissected
human islets identifies proteins and pathways potentially related
to type 1 diabetes. PLoS One.

[8] D’Souza S. S., Sri Charan Bindu B., Mohammed Ali M. (2016). Nutritional profile
of High Fat Simple Carbohydrate Diet used to induce metabolic syndrome
in C57BL/6J mice. J. Nutr. Intermed. Metab..

[9] Swarnalatha B. N., D’Souza S. S., Abraham A. (2016). Insulin resistance precedes glucose
intolerance and hyperleptinaemia in high-fat simple carbohydrate-fed
C57BL/6J mice. Endokrynol. Polym..

[10] Gangadhara V., Gawli K., Abraham A. (2026). Discrepancies
in β-adrenergic
receptor signaling and cAMP pathway in high-fat simple carbohydrate
diet-fed C57BL/6J mice: implications for metabolic syndrome pathophysiology. J. Recept. Signal Transduction.

[11] Howell S. L., Taylor K. W. (1968). Potassium ions and
the secretion of insulin by islets
of Langerhans incubated in vitro. Biochem. J..

[12] Aneesh
Kumar A., Ajith Kumar G. S., Satheesh G. (2021). Proteomics
Analysis Reveals Diverse Molecular Characteristics between Endocardial
and Aortic-Valvular Endothelium. Genes.

[13] Jing E., Sundararajan P., Majumdar I. D. (2018). Hsp90β knockdown
in DIO mice reverses insulin resistance and improves glucose tolerance. Nutr. Metab..

[14] Maiti S., Bhattacharya K., Wider D. (2023). Hsf1 and
the molecular
chaperone Hsp90 support a ‘rewiring stress response’
leading to an adaptive cell size increase in chronic stress. eLife.

[15] Rao S., Oyang L., Liang J. (2021). Biological Function
of HYOU1 in Tumors and Other Diseases. OncoTargets
Ther..

[16] Hanafusa K., Wada I., Hosokawa N. (2019). SDF2-like protein 1 (SDF2L1) regulates
the endoplasmic reticulum localization and chaperone activity of ERdj3
protein. J. Biol. Chem..

[17] Danilova T., Belevich I., Li H. (2019). MANF Is Required for
the Postnatal Expansion and Maintenance of Pancreatic β-Cell
Mass in Mice. Diabetes.

[18] Ajoolabady A., Lebeaupin C., Wu N. N., Kaufman R. J., Ren J. (2023). ER stress
and inflammation crosstalk in obesity. Med.
Res. Rev..

[19] Cheruiyot A., Hollister-Lock J., Sullivan B. (2024). Sustained hyperglycemia
specifically targets translation of mRNAs for insulin secretion. J. Clin. Invest..

[20] Lõhelaid H., Anttila J. E., Liew H. K. (2022). UPR Responsive Genes
Manf and Xbp1 in Stroke. Front. Cell Neurosci..

[21] Wang M., Wang P., Peng J. L. (2009). The altered expression
of glucose-regulated proteins 78 in different phase of streptozotocin-affected
pancreatic beta-cells. Cell Stress Chaperones.

[22] Kasai S., Kokubu D., Mizukami H., Itoh K. (2023). Mitochondrial Reactive
Oxygen Species, Insulin Resistance, and Nrf2-Mediated Oxidative Stress
Response—Toward an Actionable Strategy for Anti-Aging. Biomolecules.

[23] Bhatti J. S., Sehrawat A., Mishra J. (2022). Oxidative stress in
the pathophysiology of type 2 diabetes and related complications:
Current therapeutics strategies and future perspectives. Free Radical Biol. Med..

[24] Jiang W., Wang M., Yu X. (2025). Malignant features related
PRDX1 associated with osimertinib sensitivity of EGFR-mutant lung
adenocarcinoma. Int. J. Med. Sci..

[25] Ahlstedt B. A., Ganji R., Raman M. (2022). The functional importance of VCP
to maintaining cellular protein homeostasis. Biochem. Soc. Trans..

[26] Brusco N., Sebastiani G., Di Giuseppe G. (2023). Intra-islet insulin
synthesis defects are associated with endoplasmic reticulum stress
and loss of beta cell identity in human diabetes. Diabetologia.

[27] Penning T. M., Drury J. E. (2007). Human aldo–keto reductases:
Function, gene regulation,
and single nucleotide polymorphisms. Arch. Biochem.
Biophys..

[28] Sardelli G., Felice F., Mosca R., Avanatti M., Moschini R. (2025). Aldose Reductase
Involvement in EMT: Emerging Insights and Current Proposed Molecular
Mechanisms. Biology..

[29] Hoarau E., Chandra V., Rustin P., Scharfmann R., Duvillie B. (2014). Pro-oxidant/antioxidant balance controls
pancreatic
β-cell differentiation through the ERK1/2 pathway. Cell Death Dis..

[30] Benáková Š., Holendová B., Plecitá-Hlavatá L. (2021). Redox Homeostasis
in Pancreatic β-Cells: From Development to Failure. Antioxidants.

[31] Lee J. H., Lee J. (2022). Endoplasmic Reticulum (ER) Stress and Its Role in Pancreatic β-Cell
Dysfunction and Senescence in Type 2 Diabetes. Int. J. Mol. Sci..

[32] Good A. L., Stoffers D. A. (2020). Stress-Induced Translational Regulation Mediated by
RNA Binding Proteins: Key Links to β-Cell Failure in Diabetes. Diabetes.

[33] He Z., Liu Q., Wang Y. (2025). The role of endoplasmic reticulum stress in
type 2 diabetes mellitus mechanisms and impact on islet function. PeerJ.

[34] Um S. H., D’Alessio D., Thomas G. (2006). Nutrient overload, insulin resistance,
and ribosomal protein S6 kinase 1, S6K1. Cell
Metab..

[35] Andreadi A., Bellia A., Di Daniele N. (2022). The molecular link between
oxidative stress, insulin resistance, and type 2 diabetes: A target
for new therapies against cardiovascular diseases. Curr. Opin. Pharmacol..

[36] Fathi, I. ; Goto, M. Collagenases in Pancreatic Islet Isolation. In Transplantation, Bioengineering, and Regeneration of the Endocrine Pancreas; Academic Press, 2020; pp 529–546.

[37] Shibata A., Ludvigsen C. W., Naber S. P., McDaniel M. L., Lacy P. E. (1976). Standardization of a Digestion-filtration Method for
Isolation of Pancreatic Islets. Diabetes.

[38] Kaufman R. J., Back S. H., Song B., Han J., Hassler J. (2010). The unfolded
protein response is required to maintain the integrity of the endoplasmic
reticulum, prevent oxidative stress and preserve differentiation in
β-cells. Diabetes, Obes. Metab..

[39] Gao Y., Ryu H., Lee H., Kim Y. J., Lee J. H., Lee J. (2024). ER stress
and unfolded protein response (UPR) signaling modulate GLP-1 receptor
signaling in the pancreatic islets. Mol. Cells.

[40] Nakashima Y., Miyagi-Shiohira C., Kobayashi N., Saitoh I., Watanabe M., Noguchi H. (2017). A proteome analysis of pig pancreatic islets and exocrine
tissue by liquid chromatography with tandem mass spectrometry. Islets.

[41] Chan J. Y., Luzuriaga J., Bensellam M., Biden T. J., Laybutt D. R. (2013). Failure
of the Adaptive Unfolded Protein Response in Islets of Obese Mice
Is Linked With Abnormalities in β-Cell Gene Expression and Progression
to Diabetes. Diabetes.

[42] Claeys T., Menu M., Bouwmeester R., Gevaert K., Martens L. (2023). Machine Learning
on Large-Scale Proteomics Data Identifies Tissue and Cell-Type Specific
Proteins. J. Proteome Res..

[43] Kwon Y., Woo J., Yu F. (2024). Proteome-Scale Tissue Mapping Using Mass Spectrometry
Based on Label-Free and Multiplexed Workflows. Mol. Cell. Proteomics.

[44] Carter J. D., Dula S. B., Corbin K. L., Wu R., Nunemaker C. S. (2009). A Practical
Guide to Rodent Islet Isolation and Assessment. Biol. Proced. Online.

[45] Fröhlich K., Fahrner M., Brombacher E. (2024). Data-Independent Acquisition:
A Milestone and Prospect in Clinical Mass Spectrometry–Based
Proteomics. Mol. Cell. Proteomics.

[46] Dupree E. J., Jayathirtha M., Yorkey H., Mihasan M., Petre B. A., Darie C. C. (2020). A Critical
Review of Bottom-Up Proteomics: The Good,
the Bad, and the Future of This Field. Proteomes.

[47] Diane A., Allouch A., Mu-U-Min R. B. A., Al-Siddiqi H. H. (2024). Endoplasmic
reticulum stress in pancreatic β-cell dysfunctionality and diabetes
mellitus: a promising target for generation of functional hPSC-derived
β-cells in vitro. Front. Endocrinol..

[48] Kuzu O. F., Granerud L. J. T., Saatcioglu F. (2025). Navigating
the landscape of protein
folding and proteostasis: from molecular chaperones to therapeutic
innovations. Signal Transduction Targeted Ther..

[49] Bensellam M., Jonas J. C., Laybutt D. R. (2018). Mechanisms
of β-cell dedifferentiation
in diabetes: recent findings and future research directions. J. Endocrinol..

[50] Mladenov M., Sazdova I., Hadzi-Petrushev N., Konakchieva R., Gagov H. (2025). The Role of Reductive Stress in the
Pathogenesis of Endocrine-Related
Metabolic Diseases and Cancer. Int. J. Mol.
Sci..

[51] Chang H. H., Moro A., Takakura K. (2017). Incidence of pancreatic
cancer is dramatically increased by a high fat, high calorie diet
in KrasG12D mice. PLoS One.

[52] Assifi, M. M. ; Eibl, G. Western Diet-Induced Pancreatic Cancer. In Nutrition, Diet and Cancer; Shankar, S. ; Srivastava, R. K. , Eds.; Springer Netherlands, 2012; pp 327–338.

