# Supplementary material for: Islet Proteome Remodeling and Proteostasis Disruption in HFSC-Fed Mice
Source: ACS Omega. 2026 May 5;11(19):28391–402. doi: 10.1021/acsomega.6c00395 (PMC13191665; doi:10.1021/acsomega.6c00395)
Supplement: Supplementary file 1 [file ao6c00395_si_001.pdf]

Islet Proteome Remodeling and Proteostasis Disruption in HFSC-Fed Mice

Vijayalakshmi Gangadhara 1, Yalpi Karthik 2, Ravichandran Manisekaran *3, Asha Abraham *1.

1Father George Albuquerque Pai Cell and Molecular Biology Laboratory, Department of Biotechnology, School of Life Sciences, St Aloysius (Deemed to be University), Mangalore, Karnataka, India.

2Agrogenomic Sciences, National School of Higher Studies Unit-León, NationalAutonomous University of Mexico (UNAM), C.P. 37689 León, Guanajuato, Mexico.

3Interdisciplinary Research Laboratory (LII), Nanostructures and Biomaterials Area, Escuela, Nacional de Estudios Superiores Unidad León, Universidad Nacional Autónoma de México (UNAM), Predio el Saucillo y el Potrero, Comunidad de los Tepetates, León, C.P, 37684, Mexico.

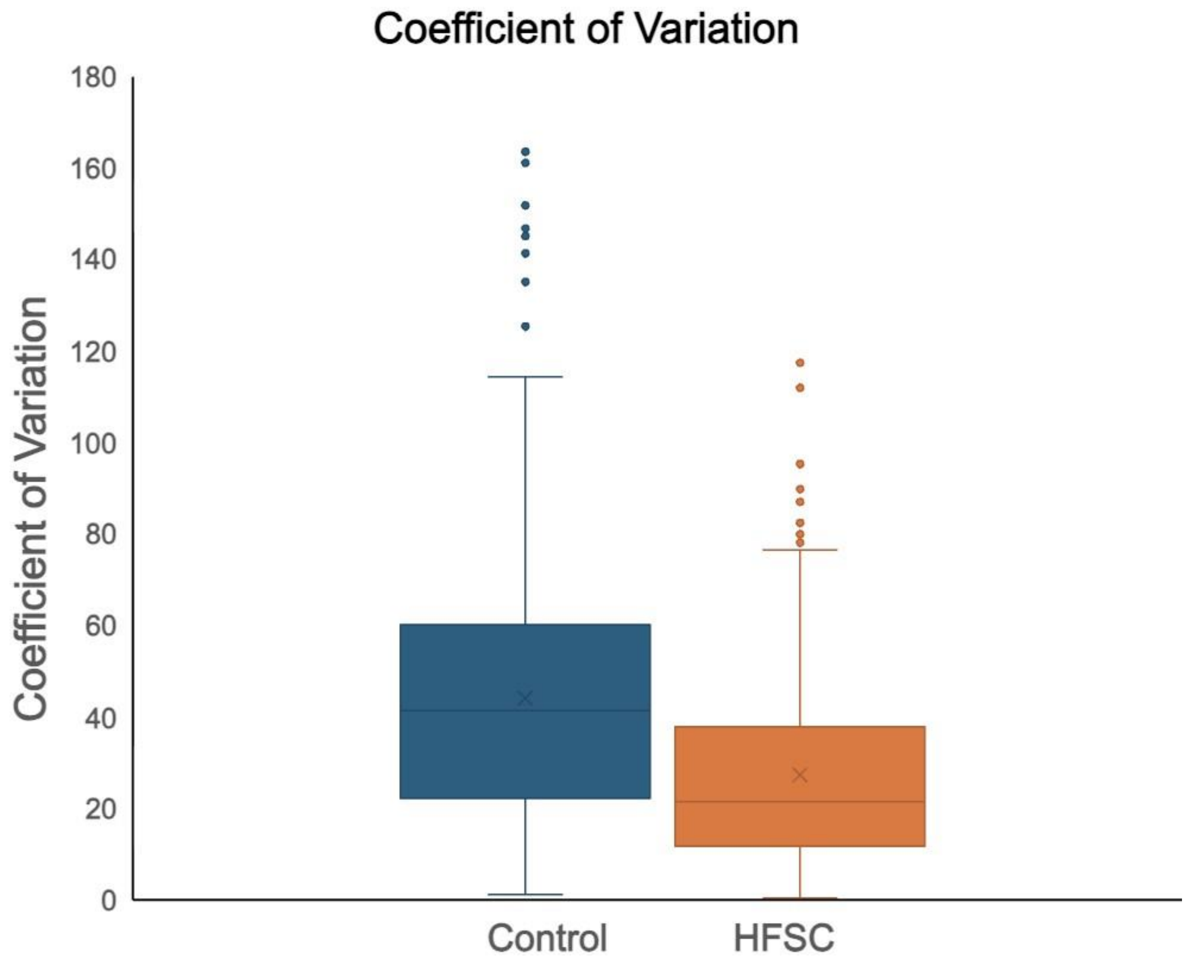
Coefficient of Variation
180
160
140
120
100
80
60
40
20
0
Coefficient of Variation
Control
HFSC

Fig. S1. Distribution of coefficient of variation (CV) values across quantified proteins in control and HFSC samples. Boxplots represent the median, interquartile range, and variability among proteins, illustrating reproducibility of protein abundance measurements across biological replicates
